# Multi-condition simulation analysis of a sliding hydraulic support system based on elastoplastic mechanics

**DOI:** 10.1038/s41598-024-61424-5

**Published:** 2024-05-09

**Authors:** Changji Wang, Wanting Wang, Yu Guo, Dewang Zhao

**Affiliations:** 1 School of Chemical and Blasting Engineering, Anhui University of Science and Technology, Huainan, 232000 China; 2 School of Mechatronics Engineering, Anhui University of Science and Technology, 168 Tai Feng Road, Huainan, 232001 China; 3 Architectural Engineering Branch, Heilongjiang Polytechnic, Harbin, 150000 China; 4 Institute of Enviromentfriendly Materials and Occupational, Anhui University of Science and Technology, Wuhu, China

**Keywords:** Coal mine in the Huainan area, Self-moving hydraulic support without repeated roof support, Elastoplastic mechanics, Numerical simulation, Engineering, Mechanical engineering

## Abstract

In response to the challenges of supporting fractured and weak surrounding rock in deep coal mines in the Huainan region of China, a self-moving hydraulic support system for roof support was designed and developed. This innovative solution addresses the difficulties encountered in providing continuous support to roof structures. Based on the theory of elastoplastic mechanics, a numerical analysis model was established to calculate the mechanical parameters such as the displacement, stress, and strain of hydraulic supports during the stepping process under multiple operating conditions. The results of the numerical simulation were compared and verified with those from an actual working site. The results show that the maximum equivalent stress is 245.33 MPa for operating condition 1, 246.82 MPa for operating condition 2, and 245.27 MPa for operating condition 3. The maximum stress values under the three working conditions do not exceed the yield strength of the material, satisfying the requirements for normal bracket support operations. These research findings can establish a theoretical framework for the comprehensive assessment of the reliability and stability of hydraulic supports and the optimization of construction processes.

## Introduction

With the gradual depletion of China's shallow coal reserves, numerous mines are transitioning towards deep mining operations. The speed and efficiency of excavation in deep rock tunnels have become important limiting factors for coal mining. The characteristics of deep geological processes and the manifestation patterns of rock pressure exhibit notable distinctions from those observed in shallow regions. To enhance tunnel excavation efficiency, ensure personnel safety, and reduce labour intensity, a crucial approach is the utilization of self-propelled support equipment in conjunction with excavation machines at the tunnel face. This enables temporary support using self-moving support equipment on newly exposed surrounding rocks subsequent to cutting by the excavation machine. This equipment can rapidly stabilize the roof of the tunnel face, thereby relocating anchor support operations from the front end to the rear end of the excavation machine. Consequently, parallel operation between tunnel excavation and anchor rod support can be achieved without interference, significantly augmenting tunnelling efficiency and facilitating safe and expeditious tunnel excavation.

Currently, control theory for coal roadway anchor supports has progressed from suspension theory, composite beam theory, and composite arch theory to surrounding rock loose circle theory and maximum horizontal stress theory. Chaoxia^1^ proposed a novel approach by integrating anchor rod support with grouting reinforcement to effectively harness the bearing capacity of surrounding rock and ensure the stability of tunnel surroundings. Zhihong et al. proposed the loose circle theory^2^. Nong^3^ and colleagues proposed the theory of low-damage continuous beam control, developed flexible anchoring technology that is not constrained by tunnel height limitations, overcame the challenges associated with complex conditions and high-density anchor rod and cable combination support technology, and established a cross-disciplinary long anchorage with a large spacing single support technique. Wanfeng^4^ adopted a comprehensive approach of full-length anchoring combined with anchor cable support while employing FLAC3 numerical simulation modelling to optimize the support parameters, thereby obtaining rationalized support parameters. Nong^5^ proposed three techniques to control the failure of U-shaped steel supports, namely, frame optimization, backfilling behind the wall, and reinforcement of weak points with anchor rods (cables). Additionally, these researchers suggested four techniques for controlling the failure of anchor rod (cable) supports, namely, strengthening the anchor rod body, enhancing the performance of the protective components, improving the resin anchoring agent performance, and implementing lag grouting in the surrounding rock. Drawing on the concept of energy consumption support for surrounding rock, Qun et al. devised a 'restrictive support resistance damper' to effectively regulate the dissipation of energy and control deformation in tunnels^6^. Zhipeng et al.^7^ proposed the active implementation of steel pipe concrete support technology in deep soft rock tunnels. Zhenqi^8^ proposed the primary research direction for investigating advanced technologies in coal mine strata control. Qi^9^ introduced the concept of advanced support, incorporating long-distance assistance and nonrepetitive aid.

The recovery roadway serves as a vital passage for safe ventilation and auxiliary transportation in the longwall comprehensive mining face, and its effective maintenance plays a pivotal role in ensuring safety and enhancing production efficiency. Due to the influence of mining activities, the surrounding rock in the tunnel ahead of the working face experiences increasing rates of damage and deformation, particularly under conditions of poor rock stability. Consequently, substantial deformations occur near the working face, thereby impacting safety protocols and compromising production efficiency at the site. Therefore, to mitigate the detrimental impact of concentrated stress ahead of mining on roadways, it is imperative to proactively reinforce the support system of the retreat pathway. Reinforcement support in fully mechanized mining faces currently relies primarily on a range of advanced support equipment. These include individual hydraulic props and various types of advanced support frames. The manual operation of a single hydraulic advance support in coal mining, due to its limitations such as high labour intensity, low support efficiency, uneven initial support strength, and certain safety hazards, has made it challenging to meet the requirements for safe and efficient advance support in working faces. Currently, numerous comprehensive mining operations employ advanced support systems to enhance stability, including integrated advanced support units, segmented advanced support units, and individual supports. Reputable scholars have extensively investigated the issue of advanced support in retreat roadways, accounting for diverse geological conditions for mining. The design principle of a “low initial support force, high working resistance” for advanced support was proposed by Wang Guofa et al.^10^ based on the analysis of the stress influence range and distribution law in the gob-side entry retaining system. This has led to the development of advanced support equipment and a roadway surrounding rock coupled support system. In response to the challenging geological conditions and support complexities encountered in a large-section roadway with an 8.2 m high mining face, Mingzhong et al.^11^ devised an intelligent self-propelled advanced support system that was successfully implemented during field trials. Kun et al.^12^ conducted an analysis on the mechanical characteristics of the coupling support system between advanced hydraulic supports and roadway anchor bolts (cables), as well as the nonuniform strength support strategy of advanced support system anchoring. This analysis was further validated through similar simulation experiments. Yajun et al.^13,14^ investigated the theory of adaptive support for advanced supports and examined the adaptive shifting method of unit-type advance support with a spiral propulsion walking mechanism. This approach effectively addresses the challenge of coordinating unit-type advance support with tunnel roofs and anchor protection systems. In response to the challenges posed by advanced support for thin and medium-thick coal seam roadways in composite roofs, Xicai et al.^15^ developed a lightweight self-moving advanced support system comprising end supports and advanced supports, which has been successfully implemented on site. The aforementioned research has facilitated the advancement of advanced support technology for roadway excavation, which has significant implications for ensuring the rational and efficient maintenance of backfill roadways within the influence area of advanced mining.

However, there is still a dearth of research and development on advanced support technology for tunnelling under complex geological conditions in the Huainan mining area, China. Considering the actual roof conditions in this region, it is imperative to avoid repetitive roof support to mitigate roof strata fragmentation and alleviate the challenges associated with maintenance work. Conventional two-column or wide-body formwork systems rely on the movement of jacks to transfer and reposition formwork. The underlying principle of this approach involves the front column supporting and pulling the rear column, while the rear column supports and pushes the front column. However, in the case of a single slab, these two columns need to repeatedly provide mutual support during the repositioning process, leading to potential damage to the slab. To address this formidable challenge, in our study, a self-propelled hydraulic support system featuring nonrepetitive roof support is designed and developed. Drawing on the principles of elastoplastic mechanics, a numerical analysis model is established to compute the mechanical parameters such as the displacement, stress, and strain of the hydraulic support system during the stepping process under various operational conditions. This comprehensive evaluation enables us to assess the reliability and stability of the hydraulic support system while providing a theoretical foundation for optimizing construction processes.

## Geological overview and resistance determination for advanced support equipment operations in the Huainan mining area

**Figure 1:**
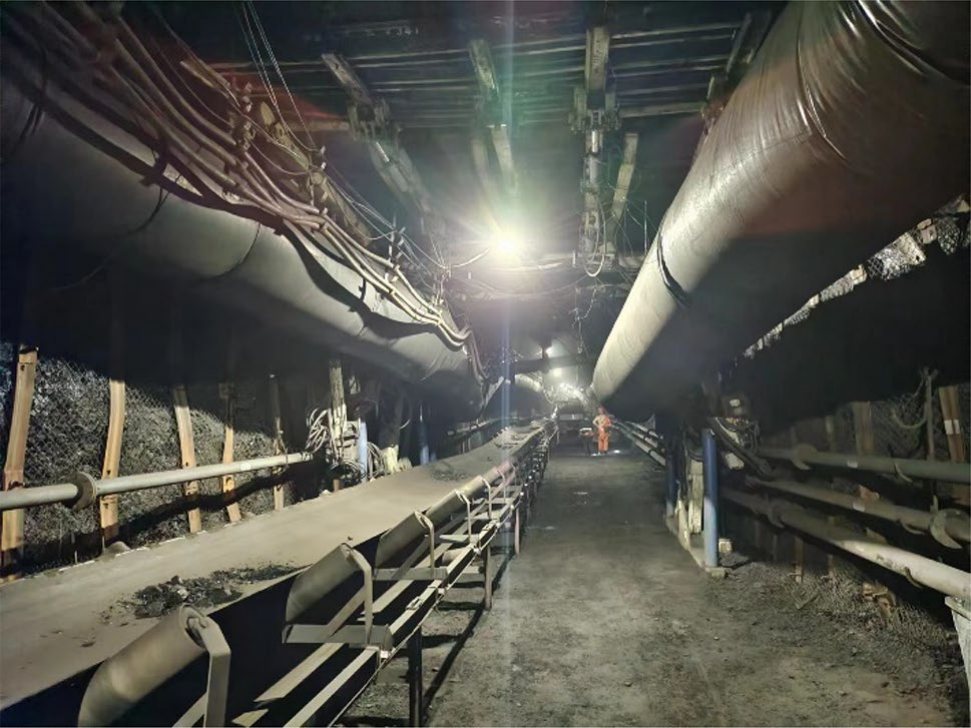
Real scene of the experimental tunnel.

The experimental roadway is located in the southern mine of Guqiao, Huainan, with a working face elevation ranging from −565.6 to −605.5 m and a ground elevation ranging from + 21.0 to + 23.3 m. The length along the direction of the working face spans from 2224.2 to 2528.3 m, with a dip length of 249 m, as depicted in Fig. 1.

According to the comprehensive analysis of field measurement data, nearby boreholes, and 3D seismic data, the stability of the 11-2 coal seam in the working face is attributed to its predominant composition of bright black coal. Additionally, intermittent bands of semi-bright coal and occasional layers of carbonaceous mudstone measuring between 0.1 and 0.4 m thick have been observed within this complex structure. According to previous research, on-site sampling was conducted to perform experimental tests on roof and coal samples, from which physical and mechanical parameters such as compressive strength, tensile strength, and elastic modulus were obtained (tensile strength of the roof: 4.26 MPa; compressive strength: 41.77 MPa; average tensile strength of coal seam 11-2: 1.23 MPa; compressive strength: 5.39 MPa). According to the exposed thickness of rock layers in the roadway, the dip angle of the exposed rock layers in the roadway, and the number and thickness of roof and floor rock layers within a range twice as wide as the roadway, along with physical and mechanical parameters and classification of surrounding rocks for each layer within this range, geological structures, hydrogeological conditions, depth of burial for the roadway, etc., a geological mechanics assessment was conducted on the surrounding rocks during longwall mining. The complex geological conditions were characterized by the following: mudstone serving as an immediate roof with coal seam 11-3 development above coal seam 11-2, leading to easy separation and fall; multiple faults exhibiting a significant displacement (6 major faults with maximum displacement up to 13.8 m); a large burial depth (nearly 630 m); a substantial influence from tectonic stress (angle between tunnel orientation and maximum principal stress of approximately 40°); and a substantial impact from subsequent mining activities overlaying previous faults.

Consistent with the mechanical criterion that the top plate support should not rupture, it is necessary to provide sufficient working resistance for the advanced supporting system during top plate support to maintain a certain range of deformation and ensure that the tensile stress on the top plate does not exceed its allowable value. According to the analysis process of support force mechanics criteria, this study aims to investigate the working resistance using a thin plate model. Based on the mechanical model of the top plate, we determined the tensile stresses in both the x- and y-directions as σ_x_ and σ_y_, respectively:

1 $$\begin{array}{*{20}c} {\sigma_{x} = - \frac{{8\pi^{2} AEz}}{{a^{2} \left( {1 - \mu^{2} } \right)}}\left[ {\sin^{2} \frac{\pi y}{b}cos\frac{2\pi x}{a} + \mu \left( \frac{a}{b} \right)^{2} sin^{2} \frac{\pi x}{a}cos\frac{\pi y}{b}} \right]} \\ {\sigma_{y} = - \frac{{8\pi^{2} AEz}}{{b^{2} \left( {1 - \mu^{2} } \right)}}\left[ {\sin^{2} \frac{\pi x}{a}cos\frac{2\pi y}{b} + \mu \left( \frac{a}{b} \right)^{2} sin^{2} \frac{\pi y}{b}cos\frac{\pi x}{a}} \right]} \\ \end{array}$$

By considering the tensile stress on the plane (i.e., top and bottom surfaces) as the focal point of investigation, Eq. (1) enables us to derive the maximum values of tensile stress along both the x and y directions on the top surface:

2 $$\begin{array}{*{20}c} {\sigma_{xmax} = \frac{{4\pi^{2} AE\delta }}{{a^{2} \left( {1 - \mu^{2} } \right)}}} \\ {\sigma_{ymax} = \frac{{4\pi^{2} AE\delta }}{{b^{2} \left( {1 - \mu^{2} } \right)}}} \\ \end{array}$$

Here $A=\frac{q{a}^{2}}{4{\pi }^{4}D[3+3{(\frac{a}{b})}^{4}+2{(\frac{a}{b})}^{2}]}$*;* and $D=\frac{E{\delta }^{3}}{12(1-{\mu }^{2})}$ . The length of the heading roof distance a (i.e., roof span) in the advanced support equipment-equipped tunnel is generally greater than the width b of the tunnel roof. Based on the analysis using Eq. (2), the stress experienced by the heading roof in the x-direction exceeds that in the y-direction. By incorporating mechanical criteria for non-rupture of the roof, this relationship can be expressed as follows:

3 $$\frac{{12qa^{2} }}{{\pi^{2} b^{2} \delta^{2} \left[ {3 + 3\left( \frac{a}{b} \right)^{4} + 2\left( \frac{a}{b} \right)^{2} } \right]}} < \sigma_{\mu }$$

4 $$q < \sigma_{\mu } \frac{{\pi^{2} b^{2} \delta^{2} \left[ {3 + 3\left( \frac{a}{b} \right)^{4} + 2\left( \frac{a}{b} \right)^{2} } \right]}}{{12a^{2} }}$$

In the equation, q0 represents the rock load exerted on the upper plate, measured in kilonewtons (kN), and F0 denotes the operational resistance of the advanced support equipment, also measured in kilonewtons (kN). According to the mechanical analysis of the roof, the tensile stress generated on the roof can be determined by calculating the difference between the rock load exerted on it and the resistance provided by advanced support equipment, as depicted in Eq. 5:

5 $$q_{0} - \frac{{F_{0} }}{a \times b} < \sigma_{\mu } \frac{{\pi^{2} b^{2} \delta^{2} \left[ {3 + 3\left( \frac{a}{b} \right)^{4} + 2\left( \frac{a}{b} \right)^{2} } \right]}}{{12a^{2} }}$$

In the equation, 'g' denotes the load sustained by the roof in an unsupported condition, measured in Newtons. According to Eq. (5), the expression for the lower limit of the resistance of advanced support equipment can be derived as follows:

6 $$F_{o} > q_{0} ab - \sigma_{\mu } \frac{{\pi^{2} b^{2} \delta^{2} \left[ {3 + 3\left( \frac{a}{b} \right)^{4} + 2\left( \frac{a}{b} \right)^{2} } \right]}}{12a}$$

According to the actual conditions of the aforementioned tunnel and considering the physical properties of the roof strata, the support provided by advance support equipment should not result in roof crushing. Therefore, we can conclude that a minimum working resistance of 488 kN is required for advanced support equipment.

## Design of a hydraulic support frame for self-moving without repeated supporting the roof of the tunnel

### Device structure design and physical diagram

**Figure 2:**
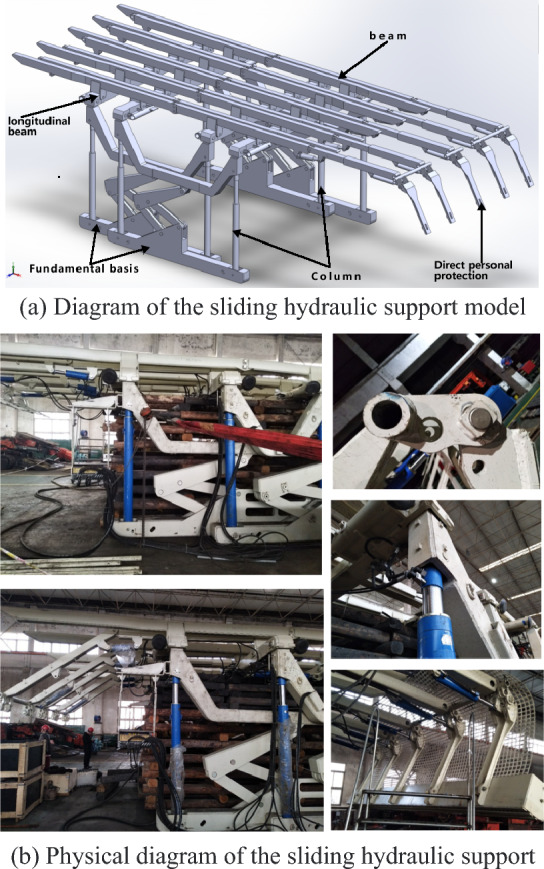
Full-scale model and physical image of the sliding hydraulic support.

Based on the actual roof conditions in the Huainan mining area and considering the geological characteristics of the experimental roadway, a sliding-type advanced support device was chosen. The device consists of a primary frame and an auxiliary frame, with the former consistently providing support to the roof during relocation. A continuous pretension force was applied to prevent detachment. The device was equipped with a base that effectively distributes pressure and prevents the bracket from causing damage to the bottom plate. The support frame was equipped with a protective head mechanism controlled by a hydraulic jack to adjust the position of the supporting head, preventing instability and collapse that could cause harm. The bracket structure was designed and modelled, and a mechanical analysis of both the bracket and self-moving system was carried out using the ANSYS and ADAMS analysis platforms. To verify the maximum load conditions of the bracket, the hydraulic bracket model is shown in Fig. 2a. The sliding temporary support device exhibits several key structural characteristics, including a primary and secondary frame structure, an alternating roof support, smooth sliding movement, and consistent pretension applied to the roof. In addition, the front and rear sections were both equipped with wire-picking mechanisms, enabling the adjustment of the network laying process based on prevailing conditions. During the relocation process, elevating the base from the ground can effectively mitigate the issue of hydraulic support base subsidence into the coal seam floor and provide structural support to the roof by incorporating a longitudinal beam, ensuring adequate space for the operation of anchor rods and cables. As a vital constituent of the rapid excavation system enabling parallel tunnelling and anchoring operations, this device functions as an intermediary support for transitioning from temporary to permanent reinforcement. Additionally, it provides a protective cover and serves as a load-bearing apparatus for laying mesh and drilling anchors. The physical hydraulic support, as per the aforementioned design scheme, is manufactured by Shenyang Tianan Technology Co., Ltd. The physical representation is illustrated in Fig. 2b.

### Analysis of the bracket structure and mobility characteristics

**Figure 3:**
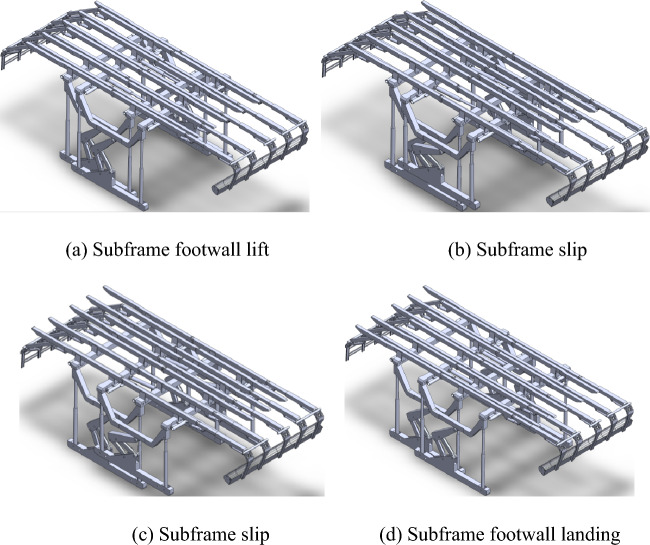
Hydraulic support model and movement diagram.

The schematic diagram in Fig. 3a–d illustrates the motion of the sliding hydraulic support. Initially, while the main frame remains in a supportive state, the horizontal and vertical beams of the auxiliary frame descend and rest on the main frame's horizontal beam. Simultaneously, as depicted in Figure (a), the bottom brace ascends. At this stage, all of the weight exerted by the auxiliary frame is borne by the main frame's horizontal beam. Subsequently, propelled by the hydraulic cylinder, the auxiliary frame ascends and accomplishes a displacement equal to the stroke of the hydraulic cylinder, as depicted in Fig. 3b,c. Subsequently, the auxiliary frame was successfully deployed, and the horizontal and vertical beams were elevated to initiate support operations. The primary frame then replicated the aforementioned movement as a mobile component, as depicted in Fig. 3d. By considering the motion and actuation characteristics of the mobile hydraulic support, the following force characteristics can be derived: (1) During the movement of the main and auxiliary frames, all the weight is exerted on the other support. In addition to hydraulic cylinder connections, there are no additional mechanical transmission structures between them. Due to frictional contact, the frictional force generated during movement may impact the stability of the supporting frame, necessitating calculation and verification. (2) During the support work, due to the uneven characteristics of the tunnel's top surface, the reverse supporting forces exerted on the supports exhibit nonuniform and nonlinear features. It is imperative to calculate and validate extreme working conditions. (3) When the main frame is in motion, potential contact between the two sides of the bracket and the sidewall of the alley may have an impact on the overall stability of the support structure.

## Establishment of a numerical model for mobile hydraulic support based on elastoplastic mechanics

Under extreme working conditions, localized plastic deformation may occur in the hydraulic support. Hence, it is imperative to establish a numerical analysis model based on elastoplastic mechanics. To comprehensively assess the reliability and stability of hydraulic supports, mechanical parameters such as displacement, stress, and strain must be calculated during the stepping process under various operating conditions. These calculations provide a theoretical foundation for optimizing construction processes.

### Finite element equation

The finite element equations for elastoplasticity were reformulated based on the principle of virtual work. The entire object was discretized into multiple finite elements, and a finite element equation for each individual element e was formulated using the principle of virtual work. The compilation of individual equations collectively constitutes the comprehensive finite element equation. For any unit e, its shape function matrix was selected as [N]. The displacement, velocity, and acceleration vectors of any point within the unit are represented as {v}, {u}, and {$\mathrm{\alpha }$}, respectively. The displacement, velocity, and acceleration vectors of the unit node are denoted as ${\{v\}}^{e},{\{{\text{u}}\}}^{{\text{e}}}$ and ${\{\mathrm{\alpha }\}}^{{\text{e}}}$, respectively. The three-dimensional problems are written as follows:

7 $$\left\{ {\begin{array}{*{20}c} {\left\{ u \right\} = \left[ {u_{1} u_{2} u_{3} } \right]^{T} } \\ {\left\{ v \right\} = \left[ {v_{1} v_{2} v_{3} } \right]^{T} } \\ {\left\{ \alpha \right\} = \left[ {\alpha_{1} \alpha_{2} \alpha_{3} } \right]^{T} } \\ \end{array} } \right.$$

8 $$\left\{ {\text{u}} \right\} = \left[ {\text{N}} \right]{*}\left\{ {\text{u}} \right\}^{{\text{e}}} { }\left\{ {\text{v}} \right\} = \left[ {\text{N}} \right]{*}\left\{ {\text{v}} \right\}^{{\text{e}}} { }\left\{ {\upalpha } \right\} = \left[ {\text{N}} \right]{*}\left\{ {\upalpha } \right\}^{{\text{e}}}$$

9 $$\left\{ {\text{b}} \right\} = \left[ {{\text{b}}_{1} {\text{ b}}_{2} {\text{ b}}_{3} } \right]^{{\text{T}}}$$

10 $$\left\{ {\text{p}} \right\} = \left[ {{\text{p}}_{1} {\text{ p}}_{2} {\text{ p}}_{3} } \right]^{{\text{T}}}$$

11 $$\left\{ {\text{q}} \right\} = \left[ {{\text{q}}_{1} {\text{ q}}_{2} {\text{ q}}_{3} } \right]^{{\text{T}}}$$

12 $$\left\{ \sigma \right\} = \left[ {\sigma_{11} \sigma_{22} \sigma_{33} \sigma_{12} \sigma_{23} \sigma_{31} } \right]^{T}$$

13 $$\left\{ {{\dot{\text{e}}}} \right\} = \left[ {{\dot{\text{e}}}_{11} {\dot{\text{e}}}_{22} {\dot{\text{e}}}_{33} { }2{\dot{\text{e}}}_{12} { }2{\dot{\text{e}}}_{23} { }2{\dot{\text{e}}}_{31} } \right]^{{\text{T}}}$$

The components $\{\dot{{{\text{e}}}_{{\text{ij}}}}\}$ in the strain rate tensor $\{\dot{{\text{e}}}\}$ at any point are defined as follows:

14 $${\dot{\text{e}}}_{ij} = \frac{1}{2}\left( {v_{i,j} + v_{j,i} } \right)$$

Based on the aforementioned equation, the matrix representation of the power flow equation for unit e can be formulated as:

15 $$\begin{aligned} \mathop \int \limits_{{V^{e} }} \left( {\left\{ {\delta v} \right\}^{e} } \right)^{T} \left[ B \right]^{T} \left\{ \sigma \right\}dV & = \mathop \int \limits_{{V^{e} }} \left( {\left\{ {\delta v} \right\}^{e} } \right)^{T} \left[ N \right]^{T} \left\{ b \right\}dV + \mathop \int \limits_{{S_{p}^{e} }} \left( {\left\{ {\delta v} \right\}^{e} } \right)^{T} \left[ N \right]^{T} \left\{ p \right\}dS \\ & \quad + \mathop \int \limits_{{S_{c}^{e} }} (\left\{ {\delta v} \right\}^{eT} \left[ N \right]^{T} \left\{ q \right\}dS - \mathop \int \limits_{{V^{e} }} \left( {\left\{ {\delta v} \right\}^{e} } \right)^{T} \left[ N \right]^{T} \rho \left[ N \right]\left\{ \alpha \right\}^{e} dV \\ & \quad - \mathop \int \limits_{{V^{e} }} \left( {\left\{ {\delta v} \right\}^{e} } \right)^{T} \gamma \left[ N \right]\left\{ v \right\}^{e} dV \\ \end{aligned}$$

16 $$\begin{aligned} \mathop \int \limits_{{V^{e} }} \rho \left[ N \right]^{T} \left[ N \right]dV\left\{ \alpha \right\}^{e} + \mathop \int \limits_{{V^{e} }} \gamma \left[ N \right]^{T} \left[ N \right]dV\left\{ v \right\}^{e} & = \mathop \int \limits_{{V^{e} }} \left[ N \right]^{T} \left\{ b \right\}dV + \mathop \int \limits_{{S_{p}^{e} }} \left[ N \right]^{T} \left\{ p \right\}dS \\ & \quad + \mathop \int \limits_{{S_{c}^{e} }} \left[ N \right]^{T} \left\{ q \right\}dS - \mathop \int \limits_{{V^{e} }} \left[ B \right]^{T} \left\{ \sigma \right\}dS \\ \end{aligned}$$

Equation (16) is the finite element equation for a single unit. By combining the individual equations, the comprehensive finite element equation can be derived as follows:

17 $$\begin{aligned} \sum \mathop \int \limits_{{V^{e} }} \rho \left[ N \right]^{T} \left[ N \right]dV\left\{ {\ddot{U}} \right\} + \sum \mathop \int \limits_{{V^{e} }} \gamma \left[ N \right]^{T} \left[ N \right]dV\{ \dot{U}\} & = \sum \mathop \int \limits_{{V^{e} }} \left[ N \right]^{T} \left\{ b \right\}dV + \sum \mathop \int \limits_{{S_{p}^{e} }} \left[ N \right]^{T} \left\{ p \right\}dS \\ & \quad + \sum \mathop \int \limits_{{S_{c}^{e} }} \left[ N \right]^{T} \left\{ q \right\}dS - \sum \mathop \int \limits_{{V^{e} }} \left[ B \right]^{T} \left\{ \sigma \right\}dV \\ \end{aligned}$$

18 $$\left[ {\text{M}} \right] = \sum \mathop \int \limits_{{{\text{V}}^{{\text{e}}} }} {\uprho }\left[ {\text{N}} \right]^{{\text{T}}} \left[ {\text{N}} \right]{\text{dV}}$$

19 $$\left[ {\text{C}} \right] = \sum \mathop \int \limits_{{{\text{V}}^{{\text{e}}} }} {\upgamma }\left[ {\text{N}} \right]^{{\text{T}}} \left[ {\text{N}} \right]{\text{dV}}$$

20 $$\sum \mathop \int \limits_{{{\text{V}}^{{\text{e}}} }} \left[ {\text{N}} \right]^{{\text{T}}} \left\{ {\text{b}} \right\}{\text{dV}} + \sum \mathop \int \limits_{{{\text{S}}_{{\text{p}}}^{{\text{e}}} }} \left[ {\text{N}} \right]^{{\text{T}}} \left\{ {\text{p}} \right\}{\text{dS}} + \sum \mathop \int \limits_{{{\text{S}}_{{\text{c}}}^{{\text{e}}} }} \left[ {\text{N}} \right]^{{\text{T}}} \left\{ {\text{q}} \right\}{\text{dS}}$$

21 $$\left[ {\text{F}} \right] = \sum \mathop \int \limits_{{{\text{V}}^{{\text{e}}} }} \left[ {\text{B}} \right]^{{\text{T}}} \left\{ {\upsigma } \right\}{\text{dV}}$$

Then, Eq. (21) can be written as:

22 $$\left[ {\text{M}} \right]\left\{ {{\ddot{\text{U}}}} \right\} + \left[ {\text{C}} \right]\left\{ {{\dot{\text{U}}}} \right\} = \left[ {\text{P}} \right] - \left[ {\text{F}} \right]$$

Equation (22) represents the general form of the finite element equation for dynamic analysis. The array $\{\ddot{{\text{U}}}\}$ represents the acceleration of all nodes in the system. $\{\dot{{\text{U}}}\}$ is the velocity array of the overall nodes, $[{\text{M}}]$ is the overall mass matrix, $[{\text{C}}]$ is the overall damping matrix, $[{\text{P}}]$ is an external force node force array, and $[{\text{F}}]$ is an array of internal force nodes.

### Yielding criterion

Considering the transition to the plastic deformation stage, we employed the Barlat–Lian yield criterion, which effectively characterizes the yielding behaviour of highly anisotropic metals as follows:

23 $${\text{f}} = {\text{a}}\left| {{\text{K}}_{1} + {\text{K}}_{2} } \right|^{{\text{M}}} + {\text{a}}\left| {{\text{K}}_{1} - {\text{K}}_{2} } \right|^{{\text{M}}} + \left( {2 - {\text{a}}} \right)\left| {2{\text{K}}_{2} } \right|^{{\text{M}}} - 2{\upsigma }_{{\text{s}}}^{{\text{M}}} = 0$$

$K_{1} = \frac{{\left( {\sigma_{xx} + h\sigma_{yy} } \right)}}{2}$, $K_{2} = \sqrt {\left( {\frac{{\sigma_{xx} - h\sigma_{yy} }}{2}} \right)^{2} + p^{2} \sigma_{xy}^{2} }$, ${\text{a}} = 2 - 2\sqrt {\frac{{{\text{r}}_{0} {\text{r}}_{90} }}{{\left( {1 + {\text{r}}_{0} } \right)\left( {1 + {\text{r}}_{90} } \right)}}}$, ${\text{h}} = \sqrt {\frac{{{\text{r}}_{0} \left( {1 + {\text{r}}_{90} } \right)}}{{\left( {1 + {\text{r}}_{0} } \right){\text{r}}_{90} }}}$, and $\frac{{2M\sigma_{s}^{M} }}{{\left( {\frac{f}{{\sigma_{xx} }} + \frac{f}{{\sigma_{yy} }}} \right)}} - 1 - \sigma_{45} = 0.$ Here ${\sigma }_{45}$ represents the tensile strength at a 45-degree angle to the direction of the load. Upon obtaining the values of variables a and h, Eq. (23) transforms into a nonlinear equation incorporating parameter p. M represents the exponent of a nonquadratic function. ${r}_{0}$ and ${r}_{90}$, represent the thick-directional anisotropy indices of two different anisotropic axes. a, h, and p represent the material parameters for anisotropic materials for which the Barlat criterion can degenerate into the Tresca yield criterion and the Mises yield criterion.

### Theory and algorithms of contact friction

During the operation of a sliding hydraulic support, there is contact between the primary and secondary frame beams, longitudinal members, and component junctions. Interactions between objects inevitably lead to relative motion and the generation of friction. Hence, when examining the force exerted during the process of sliding, precise calculations of the friction and contact forces are imperative. The calculation of frictional force was performed using the penalty function method. The calculation of the contact force employs the defense node method. The search for contact points involved the calculation and identification of all nodes in close proximity to the contacted block, which should be regarded as self-contact points. Once the contact point was determined, the contact force was primarily calculated based on the motion laws governing the interaction between the main frame and the auxiliary frame. In the penalty function method, a contact point on one surface was allowed to penetrate into another contacting surface, as shown in the following equation:

24 $${}_{{}}^{t} f_{1} = - \alpha_{1}^{t} p$$

${\alpha }_{1}$ represents the penalty function, and ${}^{t}p$ represents the boundary penetration of the contact points. The negative sign signifies that the contact force opposes the direction of boundary penetration, as it solely relies on the displacement at time **t**. Therefore, based on Eq. (24), it can be inferred that the contact force ${}^{t}{f}_{1}$ is solely dependent on the displacement at time **t**. In the defense node method, each contact pair adds a virtual contact node. The attributes of the node are identical to those of the actual node. The contact force **f** is determined by solving the motion equations of the defensive node and the coexisting contact point. The normal contact force is calculated as follows:

25 $$M_{1} a_{1} = F_{1} + f_{1}$$

26 $$M_{2} a_{2} = F_{2} + f_{2}$$

According to the central difference method, Eqs. (25) and (26) can be expressed as follows:

27 $$M_{1} \frac{{\frac{{{}_{{}}^{l} u_{1} - {}_{{}}^{\tau } u_{2} }}{\Delta t} - {}_{{}}^{l} v_{1} }}{\Delta t} = {}_{{}}^{\tau } F_{1} + {}_{{}}^{\tau } f_{1}$$

28 $$M_{2} \frac{{\frac{{{}_{{}}^{l} u_{1} - {}_{{}}^{\tau } u_{2} }}{\Delta t} - {}_{{}}^{l} v_{1} }}{\Delta t} = {}_{{}}^{\tau } F_{2} - {}_{{}}^{\tau } f_{2}$$

The normal distance between the contact point and defense point nodes is defined as:

29 $${}_{{}}^{l} g + {}_{{}}^{l} u_{2} - {}_{{}}^{\tau } u_{2} - ({}_{{}}^{l} u_{1} - {}_{{}}^{\tau } u_{1} ) = 0$$

Due to ${}^{\tau }{f}_{1}=-{}^{\tau }{f}_{2}$, according to (27), (28) and (29):

30 $${}_{{}}^{\tau } f_{1} = - {}_{{}}^{\tau } f_{2} = \left( {\frac{{{}_{{}}^{\tau } F_{2} }}{{M_{2} }} - \frac{{{}_{{}}^{\tau } F_{1} }}{{M_{1} }} + \frac{{{}_{{}}^{l} v_{2} }}{\Delta t} - \frac{{{}_{{}}^{l} v_{1} }}{\Delta t} - \frac{{{}_{{}}^{l} g}}{\Delta t}} \right)\frac{{M_{1} M_{2} }}{{M_{1} + M_{2} }}$$

${M}_{1}$ is the quality of the contact point; ${M}_{2}$ is the quality of contact nodes; ${a}_{1}$ is the normal acceleration of the contact point; ${a}_{2}$ is the normal acceleration of the defense node; ${F}_{1}$ is the normal force at the contact point node; ${F}_{2}$ is the normal force of the defense node; ${f}_{1}$ is the normal contact force at the point of contact; and ${f}_{2}$ is the normal contact force of the defense node.

In general, the calculation of friction commonly employs the conventional Coulomb's law of friction, as depicted by the following equation:

31 $$f_{j} = - \alpha_{j} u_{ij} \left( {j = 2,3} \right)$$

${f}_{2}$ and ${f}_{3}$ represent the tangential friction components.${u}_{t2}$ and ${u}_{t3}$ represent the tangential relative slip component. ${\alpha }_{1}$, ${\alpha }_{2}$, and ${\alpha }_{3}$ represent the penalty functions. If the calculated frictional force does not satisfy the pure adhesion condition, then the formula for calculating frictional force is as follows:

32 $$f_{j} = - v\alpha_{1} p\sqrt {\frac{{u_{ij} }}{{u_{t2}^{2} + u_{t3}^{2} }}}$$

### Grid partitioning and material properties

**Figure 4:**
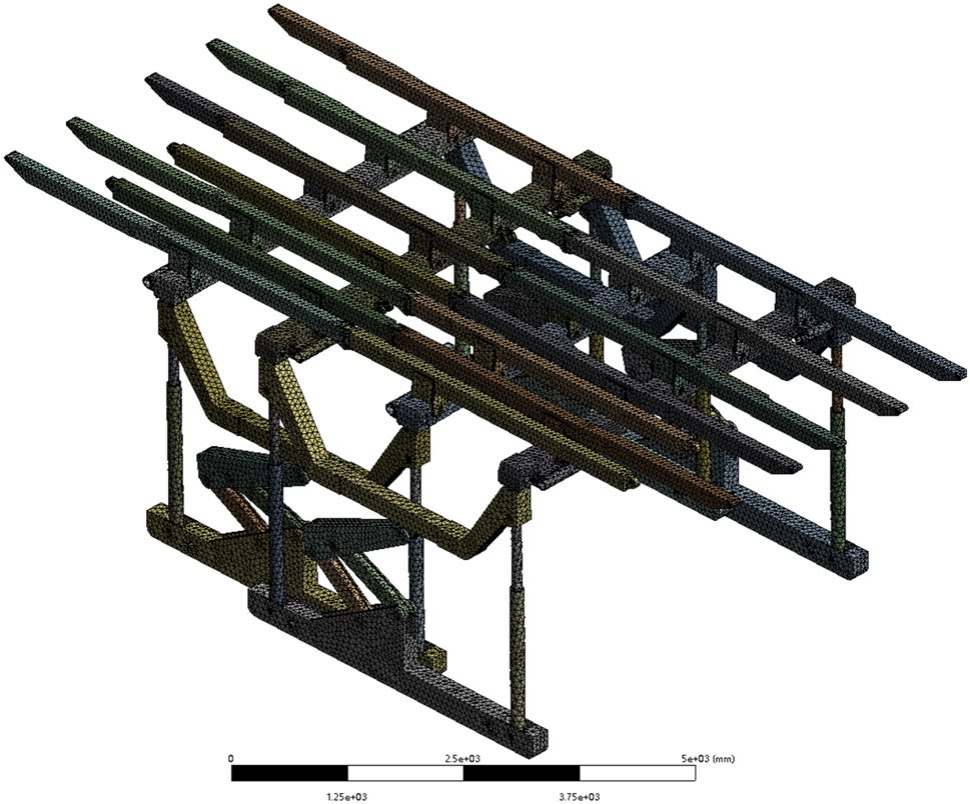
Grid division.

The hydraulic support frame with an overall structure was designed by employing a tetrahedral mesh division method. The tetrahedral mesh is a 4-node, 12-degree-of-freedom linear strain element, and its main characteristic is its high geometric adaptability. The sliding hydraulic support structure in this study is characterized by its intricate nature, featuring various complex geometries such as connection parts and supporting structures. Hence, tetrahedral elements are highly suitable for conducting this simulation study; however, discretization of critical node positions is imperative to ensure computational accuracy. Specific partitioning method: First, the overall hydraulic support model was divided into grids. The local area was further refined and encrypted to obtain a higher-quality grid. The grid division is shown in Fig. 4.

The hydraulic support was constructed with Q690 steel, a structural material that boasts exceptional low-temperature performance, plasticity, and welding properties. The material properties are shown in Table 1.

**Table 1:** Structural material properties of the hydraulic support.

Material	Density/kg m^−3^	Modulus of elasticity/GPa	Poisson's ratio	Fatigue limit/MPa
Q690	7850	200	0.3	690

### Working conditions and boundary conditions

Based on the static equilibrium equation between the hydraulic support resistance and roof strata self-bearing structure, the static load exerted by the roof strata on the hydraulic support was calculated. By incorporating an additional dynamic load coefficient, the optimal working resistance required for hydraulic support was determined to be 488 kN. To verify the stability of the new sliding hydraulic support under normal working conditions, its force characteristics under extreme conditions were analysed, and a theoretical basis for optimizing the hydraulic support and mechanized construction process of face protection was provided. Force calculations were conducted for three scenarios, including single-group supports, overall supports, and the stepping process of overall supports. Additionally, a reasonable working resistance of 488 kN was determined by applying an appropriate dynamic load coefficient to the hydraulic support. For the specific operating conditions and boundary conditions, please refer to Table 2.

**Table 2:** Working conditions and boundary conditions.

	Model diagram	Boundary condition
Operating condition 1 Single bracket		The base of the bracket is fixed with constraints, and 5 parallel beams on one side bear a load of 0.1452 MPa
Operating condition 2 Support integral		The base of the support bracket is fixed with constraints, and the longitudinal beam bears a uniformly distributed load of 0.0789 MPa
Working condition 3 Stent overall step process		The support base is fixed with constraints, and the crossbeam bears a uniformly distributed load of 0.0789 MPa. The two pushing jacks are subject to the horizontal bearing load of 32.884 kN, and the horizontal step distance of the support is 1000 mm

### Analysis and discussion of the calculation results

#### Statistical analysis was performed on a single group of scaffolds

**Figure 5:**
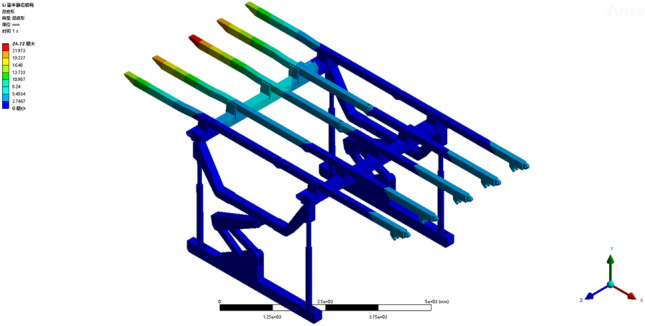
Total deformation of the monomer support under working condition 1.

**Figure 6:**
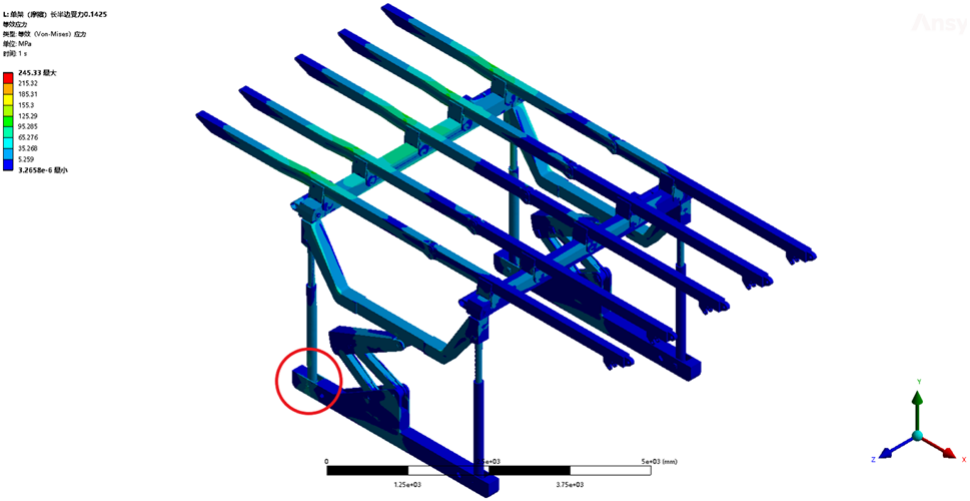
Equivalent stress cloud image of the monomer support under working condition 1.

Operating condition 1: The fixed support base, featuring five parallel beams on one side, sustains a load of 0.1452 MPa. From the deformation cloud map and stress cloud map of operating condition 1, the maximum deformation is 24.72 mm. The maximum deformation is observed at the terminus of the third straight beam, as illustrated in Fig. 5. The maximum equivalent stress is 245.33 MPa, occurring at the pin joint connecting the column and the base, as shown in Fig. 6. The maximum stress of a single set of brackets under working condition 2 does not exceed the yield strength of the Q690 material, thereby satisfying the requirements for normal support operations.

#### Statistical analysis of the whole scaffold

**Figure 7:**
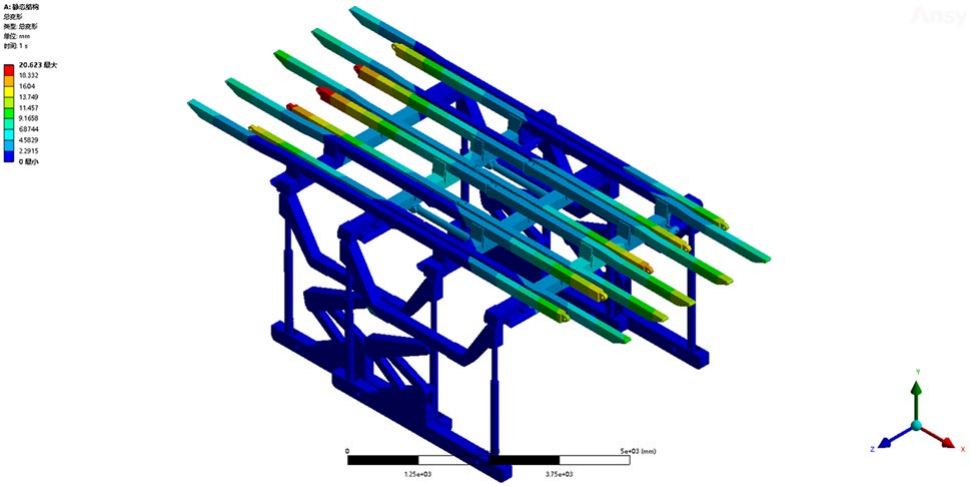
Total deformation of the whole support under working condition 2.

**Figure 8:**
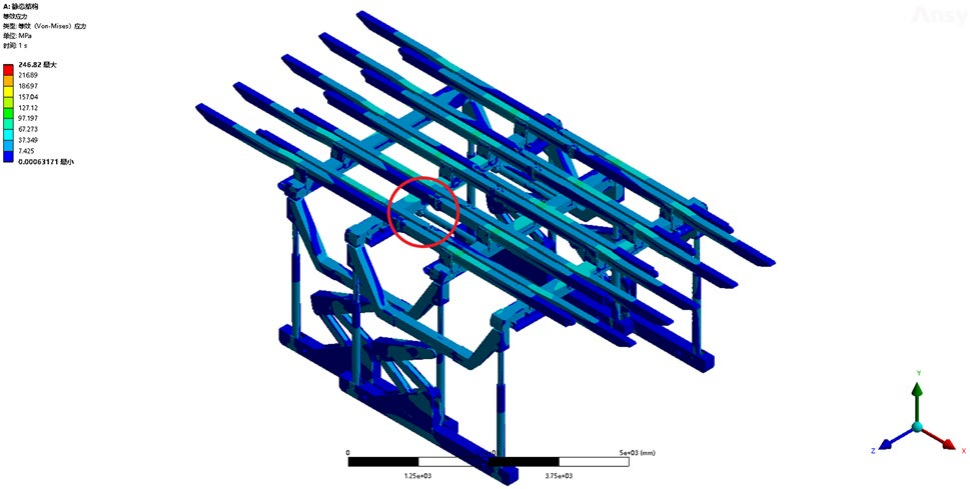
Equivalent stress cloud image of the whole support under working condition 2.

Operating condition 2: The fixed support base is capable of withstanding a uniformly distributed load of 0.0789 MPa along the beam. From the deformation cloud map and stress cloud map of the operating conditions, the maximum deformation is 20.623 mm. The maximum deformation is observed at the termination of the sixth straight beam, as shown in Fig. 7. The maximum equivalent stress of 246.82 MPa is observed at the pin shaft connecting the jacks of two sets of supports and the crossbeam, as depicted in Fig. 8. The maximum stress experienced by the overall bracket under working condition 2 remains within the yield strength of the Q690 material, thereby satisfying the requirements and enabling normal support operations for the entire bracket.

#### Statics analysis of the overall step process of the support (only one set of supports provides support)

**Figure 9:**
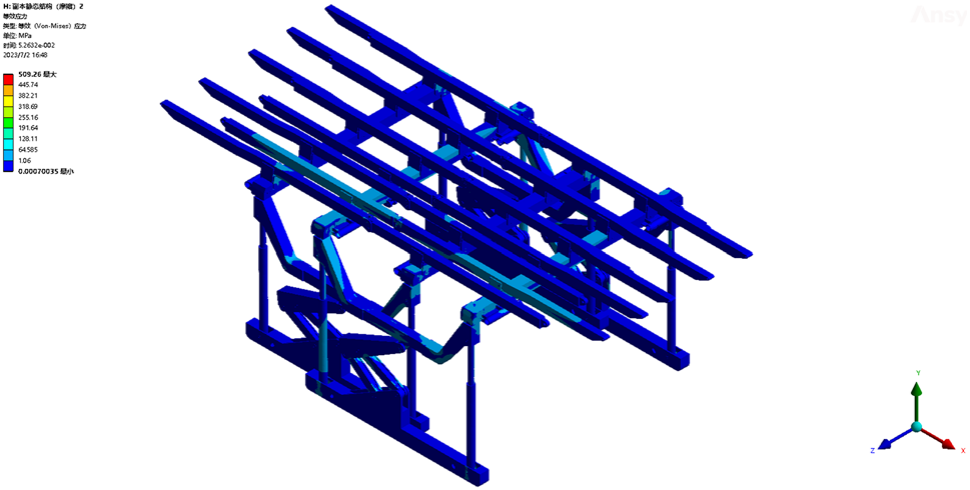
Stress change cloud map of the initial stage of the support step.

**Figure 10:**
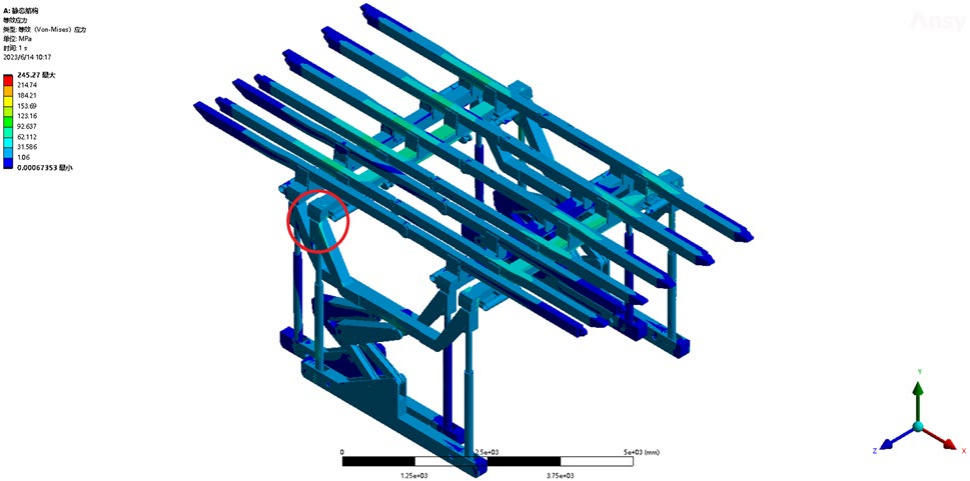
Stress change cloud map at the end of the support step stage.

**Figure 11:**
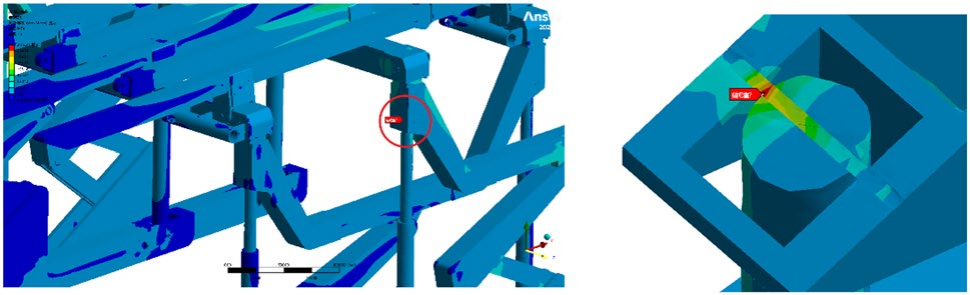
Maximum stress of the support at the step stage.

Operating condition 3: Fixed support base, with the beam bearing a uniformly distributed load of 0.0789 MPa. The two pushing jacks are subjected to a horizontal bearing load of 32.884 kN, and the horizontal step distance of the support is 1000 mm. Throughout the duration of step motion under operating condition 1, notable variations in the equivalent stress are observed, with a maximum value recorded at 245.27 MPa, as shown in Fig. 9. The maximum equivalent stress occurs at the pin joint connecting the bracket beam and column, as shown in Figs. 10 and 11. According to the yield strength of the Q690 steel, the maximum stress during the overall stepping process of the bracket (with only one set of brackets providing support) under working condition 3 does not exceed the yield strength of the material. This requirement is met, and the bracket as a whole can perform supporting operations normally.

Operating condition 1 is an analysis of the extreme force situation on a single support bracket, where the load is applied as a concentrated force on one side of the beam. In practical usage, such extreme conditions have a relatively low probability of occurrence. The hydraulic support structure demonstrates exceptional rationality, ensuring that even under extreme working conditions, the maximum stress remains well below the material's yield stress. The overall structure falls within the elastic range, indicating that the individual support structure exhibits high stability and robust reliability. A comparison between operating conditions 2 and 3 reveals that the maximum equivalent stress exhibits a similar magnitude, approximately 246 MPa. The dynamic structure and stability of the mobile hydraulic support are reasonable, as the loads generated by the movement and contact of the auxiliary frame during transportation do not significantly affect the overall stability of the structure. Through comparative analysis, it has been determined that the primary locations for maximum stress and deformation are at the beam ends, hydraulic columns, and node connections. The primary factor is as follows: The bracket primarily consists of a composite structure comprising columns, crossbeams, and longitudinal beams. When the beam span is too long, it causes large deflection deformation under load. The horizontal beam further transfers the load to the vertical beams and columns, causing a certain amount of deformation in the columns. Node connections inevitably lead to stress concentration. The structural connections proposed in this study are all pin connections with frictional contact, which further increases the stress level. To enhance the overall stability and reduce the maximum equivalent stress, further improvements can be implemented on the bracket from two perspectives. First, the number of beam sections can be increased to enhance the overall structural stiffness and reduce stress levels by transforming the two-section beams into multiple sections. The connection method can be further improved.

## Experimental situation

**Figure 12:**
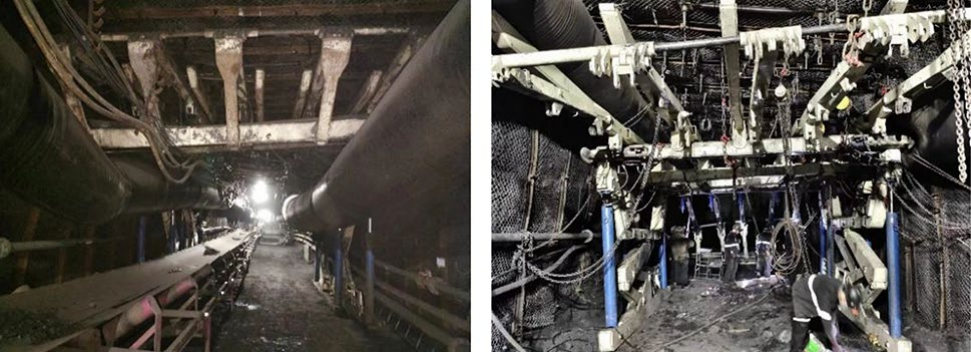
Field operation diagram.

The slip-type hydraulic support designed and developed in this study was manufactured by the Shenyang Tianan Technology Company. The safety support operation for the upper test roadway in the South II Upper Panel Area of Guqiao Nan Mine in Huainan was successfully completed, spanning a total length ranging from 2224.2 to 2528.3 m. The on-site operation diagram is shown in Fig. 12. During the assignment process, the maximum deformation of the hydraulic support bracket aligns consistently with the simulation results, which are observed at both ends of the beam and its nodes. The simulation results demonstrated the accuracy, efficiency, and safety of the scaffold, thereby establishing a computational model foundation for further structural optimization.

## Conclusions

In this study, a working resistance of 488 kN is determined by conducting a mechanical analysis and calculation of a tunnel roof, taking into account the actual geological conditions of the Huainan mining area in China. A self-moving hydraulic support bracket without a repeated support roof is designed and developed, and its movement characteristics are analysed. A numerical analysis model based on the theory of elastoplastic mechanics is developed to calculate the mechanical parameters, including the displacement, stress, and strain of hydraulic supports during the stepping process under various operating conditions. The research findings reveal that the maximum equivalent stress and displacement under operating condition 1 are 245.33 MPa and 24.72 mm, respectively. The maximum equivalent stress is 246.82 MPa, and the maximum displacement is 20.623 mm under operating condition 2. The maximum equivalent stress under operating condition 3 is 245.27 MPa. The maximum stress values under all three working conditions do not exceed the yield strength of the material, meeting the requirements for normal operation of the support bracket. The influence of local stress concentration induced by constraints can be disregarded in the analysis of static strength phenomena. Revisions to the connection method can be implemented to optimize the overall stability of the bracket during movement and to minimize the maximum equivalent stress.

## Data Availability

All data generated or analysed during this study are included in this article.
